# Serum calprotectin can indicate current and future severity of COVID‐19

**DOI:** 10.1002/jcla.24809

**Published:** 2022-12-16

**Authors:** Hajar Shokri‐Afra, Mona Moradi, Hadis Musavi, Hemen Moradi‐Sardareh, Bahman Moradi poodeh, Arash Kazemi Veisari, Ziaeddin Oladi, Mahboobe Ebrahimi

**Affiliations:** ^1^ Gut and Liver Research Center, Non‐communicable Diseases Institute Mazandaran University of Medical Sciences Sari Iran; ^2^ Pediatric Infectious Diseases Research Center, Communicable Diseases Institute Mazandaran University of Medical Sciences Sari Iran; ^3^ Department of Clinical Biochemistry, School of Medicine Babol University of Medical Sciences Babol Iran; ^4^ Department of Research and Technology Asadabad School of Medical Science Asadabad Iran; ^5^ Biomad company Oslo Norway; ^6^ Department of Laboratory Sciences, Lahijan Branch Islamic Azad University Lahijan Iran; ^7^ Department of Internal Medicine, School of Medicine, Ghaem Shahr Razi Hospital Mazandaran University of Medical Sciences Sari Iran

**Keywords:** calprotectin, COVID‐19, IL‐6, inflammatory biomarkers, outcome

## Abstract

**Background:**

Predictive and prognostic biomarkers to guide 2019 novel coronavirus disease (COVID‐19) are critically evolving. Dysregulated immune responses are the pivotal cause of severity mainly mediated by neutrophil activation. Thus, we evaluated the association of calprotectin, neutrophil secretory protein, and other mediators of inflammation with the severity and outcomes of COVID‐19.

**Methods:**

This two‐center prospective study focused on PCR‐proven COVID‐19 patients (*n* = 76) with different clinical presentations and SARS‐CoV‐2 negative control subjects (*n* = 24). Serum calprotectin (SC) was compared with IL‐6 and other laboratory parameters.

**Results:**

Median levels of SC were significantly higher in COVID‐19 patients in comparison to the control group (3760 vs. 2100 ng/ml, *p* < 0.0001). Elevated SC was significantly respective of disease severity (3760 ng/ml in mild up to 5700 ng/ml in severe cases, *p* < 0.0001). Moreover, the significant positive and negative correlations of SC with disease severity and oxygenation status indicated disease progression and respiratory worsening, respectively. It was found that SC was high in severe patients during hospitalization and significantly declined to normal after recovery. The logistic analysis identified the independent predictive power of SC for respiratory status or clinical severity. Indeed, SC behaved as a better discriminator for both outcomes, as it exhibited the largest area under the curve (receiver operating curve analysis), with the highest specificity and sensitivity when the predictive value of inflammatory biomarkers was compared.

**Conclusion:**

Calprotectin can be used as a reliable prognostic tool to predict the poor clinical outcomes of COVID‐19 patients.

## Graphical abstract

SC and IL‐6 had a disease severity‐dependent response for COVID‐19 patients. However, SC content exhibited a better‐differentiated pattern in which it increased significantly with the exacerbation of disease clinical symptoms. Mild to severe subgroups had significantly different SC and IL‐6 levels, regardless of how many days had elapsed since the disease onset. Indeed, SC and IL‐6 levels were not affected by the time of sampling indicating a contiguous inflammatory status in patients because inflammatory markers decreasing reflects attenuating inflammation and consequently an improvement in disease severity. As we observed that SC and IL‐6 levels significantly reduced to normal in recovered patients and were comparable to control cases. Of note, their decrease was respective of disease severity. In general, SC was found as a more reliable marker than IL‐6 in the COVID‐19 investigation.

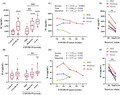

## 1 INTRODUCTION

COVID‐19 has been introduced as a highly inflammatory disease that excessive inflammatory response to SARS‐CoV‐2 seems to be the major cause of the disease severity.^1^ Evidence expressed the ability of SARS‐CoV‐2 to enter respiratory epithelial cells, where it proliferates, causes acute inflammation, and worsens the clinical features of disease by producing proinflammatory cytokines.^2^, ^3^ Dysregulation of the immune system and cytokine storm has been closely associated with the immunopathogenesis of this virus^4^ causing complications, including pneumonia, loss of lungs function, as well as acute respiratory distress syndrome (ARDS), shock, and even death.^5^, ^6^ Neutrophils and macrophages are major sources of proinflammatory cytokines.^7^, ^8^ Studies that investigated COVID‐19 pathophysiology reported an elevated level of macrophage markers (IL‐6 and IL‐1*β*), as well as higher levels of blood neutrophils associated with poor prognosis in COVID‐19 patients.^9^, ^10^ However, there are ongoing reports for alternative biomarkers that may serve as better indicators for COVID‐19 severity^11^, ^12^ that needs to be fully recognized.

Calprotectin is a heterocomplex of two calcium‐binding protein subunits S100A8 and S100A9, produced by the immune system, mainly neutrophils, and at lower levels of monocytes and/or macrophages, platelets, and epithelial cells.^13^ These two subunits make up approximately 45% of the cytoplasmic proteins present in neutrophils.^14^ Calprotectin acts as a ligand for pattern recognition receptors, such as Toll‐like receptor 4 (TLR‐4), and receptor for advanced glycation end products (RAGE), which mediates the production of pro‐inflammatory cytokines via inducing neutrophils and macrophages migration to inflammation sites.^15^ Therefore, calprotectin contributes to homeostasis during inflammation, but its overexpression and secretion make an imbalance in inflammatory processes.^14^ Calprotectin increases in infectious, as well as in a wide variety of inflammatory and autoimmune conditions (e.g., inflammatory bowel disease (IBD), and rheumatoid arthritis (RA)) where it can be considered as a marker for diagnostic purposes. Additionally, serum calprotectin (SC) was recognized as a powerful marker to determine the disease activity, response to treatment, and monitoring the remission or recurrence of inflammatory diseases.^16^, ^17^

To date, several biomarkers, including raised C‐reactive protein (CRP), erythrocyte sedimentation rate (ESR), ferritin, and IL‐6, have been proposed to evaluate severe and non‐severe COVID‐19.^18^, ^19^ However, an elevated calprotectin accompanied by neutrophil activation has been suggested as a robust biomarker of COVID‐19 severity.^11^, ^12^ Nevertheless, little is yet known about how neutrophil activation is implicated in COVID‐19, which may dispel our ambiguities about COVID‐19 inflammatory status. We measured the SC of hospitalized COVID‐19 patients and determined its association with COVID‐19 severity, respiratory status, disease prognosis, and most importantly patients' recovery in comparison with some other confirmed biomarkers.

## 2 METHODS AND MATERIALS

### 2.1 Patients

This was a two‐center prospective cohort study, performed at Razi Hospital and Imam Khomeini Hospital, the two main centers of COVID‐19 in Mazandaran province, Iran. Following the 9th Edition guidelines of the Ministry of Health and Medical Education,^20^ SARS‐CoV‐2 infection confirmation was based on positive qPCR results for SARS‐CoV‐2 RNA and clinical symptoms with chest CT examination findings related to COVID‐19. The COVID‐19 group was selected from inpatients who met the above criteria. The control group were asymptomatic inpatients with negative qPCR results for SARS‐CoV‐2 who voluntarily participated in the study (non‐COVID‐19 subjects). They were selected nearly the same age and gender as COVID‐19 patients. All participants enrolled during September and November 2020 and provided informed consent to participate in the study. However, patients younger than 18 years old, patients without informed consent, as well as patients with inflammatory bowel disease and/or autoimmune associated conditions such as celiac disease, lupus, and rheumatoid arthritis were excluded. Finally, a total of 100 subjects (76 COVID‐19 and 24 non‐COVID‐19) were enrolled in the current study.

This study was approved by the Ethics Committee of Imam Khomeini Hospital, Mazandaran University of Medical Sciences (IR.MAZUMS.IMAMHOSPITAL.REC.1399.035). All methods were carried out in accordance with relevant guidelines and regulations.

### 2.2 Study definitions

According to the WHO‐China Report for COVID‐19,^21^ inpatients were stratified into three categories based on disease severity during admission: mild disease (*n* = 30) was defined as having mild clinical symptoms with limited lung involvement (<1/3) and SpO_2_ > 93%; moderate disease (*n* = 25) was defined as more symptomatic with less than 50% radiological findings on chest CT‐scan, and SpO_2_ 90%–93%; severe disease (*n* = 21) was defined as respiratory distress with more than 50% lung involvement and SpO_2_ < 90% that may require mechanical ventilation or intensive care unit (ICU) admission. A chest CT scan was performed to determine this classification before admission. Demographic/clinical data, information on comorbidities, disease symptoms, dates of onset, hospital admission, laboratory parameters, and discharge, or death were recorded through interviews and patients' medical records. Patients' personal information was kept confidential and researchers analyzed only anonymous data.

Oxygenation status and clinical respiratory status were considered criteria for patients' clinical status in addition to the disease severity. The oxygenation status was considered adapted from hemoglobin (Hb) and oxygen saturation (SpO_2_). Respiratory status was considered based on how patients received oxygen, mechanically or noninvasively. In this regard, two conditions were considered as noninvasive oxygen support such as nasal‐cannula (flow of 2–5 L/min) or face mask (flow of 5–9 L/min). The length of hospitalization period also reflected the disease severity.

### 2.3 Sampling and laboratory examinations

Blood sampling was performed during admission and/or hospital stay (mainly in the first 2 d after admission), but 20 patients had sera available after 6.60 (± 2.04) days of their hospitalization (3 mild, 10 moderate, and 7 severe) when treatment had been started in these cases. It can be mentioned from another aspect that clinically, all patients were sampled when they were at the peak of their illness. Therefore, sampling was repeated for 8 recovered patients before discharge for more investigation.

Whole blood was collected in EDTA tubes for complete blood count (CBC), and anticoagulant‐free biochemistry tubes were used for serum separation. Part of the collected serum was used for all laboratory tests performed in the hospitals' laboratories, following the standard procedures. The other part of serum was stored at −80°C for further analysis of SC and IL‐6 quantification. Before analysis, samples were carefully brought to room temperature (15–18°C) and homogenized before use. SC levels were measured by sandwich ELISA method via the CALPROLAB™ Calprotectin ELISA (HRP) (CALPRO AS) according to the manufacturer's protocol. The lower detection limit of the kit was <5 ng/ml, and the mean CV was 7%. IL‐6 was quantified using the ELISA kit KPG‐HI6 (Karmania Pars Gene), according to the manufacturer's instructions. In the study conducted by the manufacturer, the sensitivity of this kit was 3 pg/ml and intra‐ and inter‐assay accuracy were <3% and <9%, respectively.

### 2.4 Statistical analysis

Descriptive statistics were applied to summarize the demographic data. Results are reported as frequency or medians or means with standard deviations. Kruskal–Wallis test was used to identify statistical differences between COVID‐19 subgroups. Continuous variables were compared between groups using Student's *t*‐test and the Mann–Whitney *U* test. Chi‐square test was performed to compare categorical variables. Associations between parameters were calculated using Spearman's correlation. Linear regression model estimated the effect relationship of symptoms onset time with biomarkers. The interrelationship of the two variables of severity and symptoms onset on biomarkers was assessed by two‐way analysis of variance (ANOVA). Logistic regression was performed to explore the risk factors associated with the severity and invasive ventilation. The effect of confounding variables was identified using univariate logistic regression analysis and nonsignificant variables (*p*‐Values more than 0.2) were eliminated for the multivariate logistic regression analysis. Receiver operating curve (ROC) analysis was used to further evaluate the potential clinical utility of indicated laboratory parameters as predictive biomarkers for invasive ventilation and disease severity. Statistical Package for Social Sciences (SPSS) 22.0 software (IBM) and GraphPad Prism (version 9) (GraphPad, Inc) were used for statistical analysis and illustrations. Statistically significant values are as *p* < 0.05.

## 3 RESULTS

### 3.1 Patients' characteristics

As shown in Table 1, COVID‐19 patients in the severe group were significantly older than the moderate and mild groups (*p* = 0.047). Body mass index (BMI) was not different between COVID‐19 males (24.5) and females (28.4) (*p* = 0.116) as well as between disease categories (*p* = 0.605). Comorbidities were observed more in the severe group, ranging from 70% in the mild group to 85.7%. Diabetes, hypertension and cardiovascular disease were the most common comorbidities in the COVID‐19 group. None of the COVID‐19 patients were drug or opium users and also had no history of chronic obstructive pulmonary disease (COPD). We found that symptoms onset until hospitalization time varied for each patient. Severe patients were hospitalized much later, and blood sampling was subsequently performed later (12th day). In addition, severe patients had to be treated significantly longer than mild and moderate patients (*p* = 0.005). Death occurred only in the severe group (19%). COVID‐19 and the control groups were age‐, sex‐, and coexisting disorders‐matched. However, COVID‐19 patients had significantly higher BMI than the control group (*p* = 0.001).

**TABLE 1:** Baseline characteristics of inpatients enrolled.

	COVID‐19 subgroups				Groups		
Characteristics	Mild *n* = 30 (39.5%)	Moderate *n* = 25 (32.9%)	Severe *n* = 21 (27.6%)	Sig.*	COVID‐19 *n* = 76	Control *n* = 24	Sig.#
In years (± SD)	46.7 (± 15.5)	53.2 (± 18.9)	58.6 (± 15.9)	**0.047**	52.1 (± 17.2)	52.83 (± 22.61)	0.870
≥ 65 years (%)	3 (10)	8 (32)	8 (38.1)	0.268	19 (25)	8 (33.3)	0.553
Gender, Male (%)	14 (46.7)	14 (56)	11 (52.4)	0.794	39 (51.3)	16 (66.7)	0.188
BMI (P25‐P75)	26.8 (22.4–29.4)	25.8 (23.4–28.8)	27.8 (24.2–32.5)	0.605	26.8 (23.3–29‐4)	22.77 (± 4.21)	**0.001**
BMI ≥30 (%)	6 (20)	1 (4)	5 (23.8)	0.174	12 (15.8)	1 (4.17)	0.121
**Coexisting disorders** (%)	21 (70)	17 (68)	18 (85.7)	0.334	56 (73.7)	17 (70.8)	0.784
Diabetes	8 (26.7)	6 (24)	11 (52.4)	0.081	25 (32.9)	4 (16.7)	0.127
Hypertension	7 (23.3)	8 (32)	9 (42.9)	0.336	24 (31.6)	4 (16.7)	0.156
Cardiovascular disease	7 (23.3)	4 (16)	4 (19)	0.790	15 (19.7)	2 (8.3)	0.195
Chronic kidney disease	2 (6.7)	1 (4)	5 (23.8)	0.063	8 (10.5)	3 (12.5)	0.788
Chronic liver disease	3 (10)	2 (8)	2 (9.5)	0.966	7 (9.2)	1 (4.2)	0.427
**Hospitalization characteristics**							
Symptoms onset^&^, days	8 (5–12)	9 (6–13)	12 (8–16)	0.130	9 (6–13)	‐	‐
Hospitalization period, days	6 (5–7)	7 (6–10.5)	9 (7–13)	**0.005**	7 (6–10)	4 (3–5.7)	**<0.0001**
Mortality rate (%)	0	0	4 (19)	‐	4 (5.3)	0	‐
**Laboratory parameters**							
NEU (×10^3^/μl)	3.9 (2.8–5.6)	4.6 (3.5–6.4)	6.4 (4–7.9)	**0.018**	4.6 (3.5–6.8)	4.9 (2.9–6.2)	0.620
NEU%	68.5 (9.6)	70.3 (9.3)	72.9 (5.9)	0.220	70.3 (8.7)	63 (6.3)	**0.0006**
LYM (×10^3^/μl)	1.4 (1–1.8)	1.2 (0.9–1.9)	1.4 (1–1.8)	0.949	1.4 (1–1.8)	2.2 (1.7–2.7)	**<0.0001**
LYM%	24.1 (8.9)	20.8 (7.4)	17.8 (5.3)	**0.017**	21.3 (7.9)	28.4 (7)	**0.0003**
NLR	2.5 (1.9–4.6)	3.6 (2.4–4.8)	4.2 (3–5.4)	**0.047**	3.6 (2.3–4.7)	2.3 (1.6–2.7)	**0.0005**
PLT (×10^3^/μl)	168 (138–206)	189 (160–240)	140 (122–237)	0.404	172.5 (137–206)	249.5 (209–324)	**<0.0001**
LDH (U/L)	461 (384–667)	554 (396–706)	694 (619–917)	**0.009**	572 (418–721)	327 (251–379)	**<0.0001**
ESR (mm/h)	41.2 (21)	39.1 (21.2)	49.5 (16.1)	0.302	42.9 (19.9)	26.6 (11.8)	**0.002**
CRP (mg/dl)	32.6 (11.8–45)	33.5 (16.3–36)	38 (32–49)	0.056	35 (23–39.5)	6.3 (2.8–21.4)	**<0.0001**

*Note*: Data are expressed as mean (± SD) or frequency (percentage) or median (25th‐75th percentiles). The statistically significant difference with a *p*‐value less than 0.05 is shown in bold.

* The comparison between the three COVID‐19 subgroups (Kruskal–Wallis test).

^**#**^ The comparison between the COVID‐19 and control groups (Student's *t*‐test for normal and the Mann–Whitney *U* test for non‐normal variables). & The interval time between symptoms onset and the time of sampling.

### 3.2 Serum calprotectin and IL‐6 relation to COVID‐19 clinical status

**FIGURE 1:**
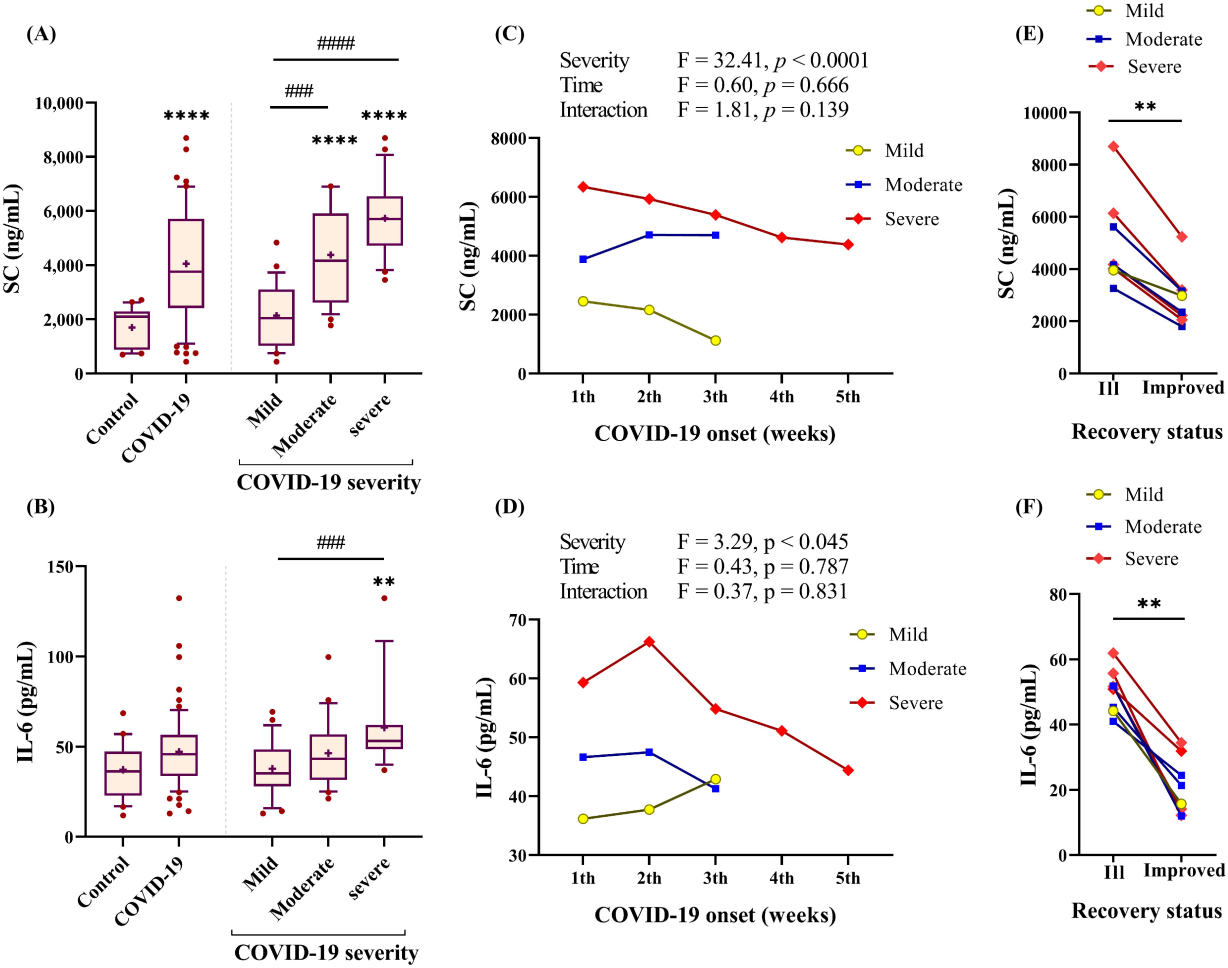
SC and IL‐6 association with COVID‐19 severity. (A, B) Serum from control subjects (*n* = 24) and COVID‐19 patients (*n* = 76) with three severity forms were assessed for SC and IL‐6 evaluation. Plots are displaying the 25–75 percentiles at the boxes, the median in the center line, the 10–90 percentile at the whiskers, and means as + in each column. A post hoc test, Dunn's multiple comparisons test, was used after a significant Kruskal–Wallis test; all columns were compared to control: **p < 0.01, ****p < 0.0001, and severity columns were compared pairwise: ##*p <* 0.001, *####p* < 0.0001 shown as a line. (C, D) The main effects and interactions of COVID‐19 severity and the time of disease onset (or sampling) on SC and IL‐6 levels were evaluated by two‐way ANOVA. Line graphs illustrate medians. (E, F) For 8 patients, serum samples were available from two‐time points when patients were ill or improved; **p < 0.01 by paired Wilcoxon test.

Our results showed an increase in SC levels of COVID‐19 patients compared with control subjects (3760 vs. 2100 ng/ml, *p* < 0.0001). IL‐6 also showed higher levels in COVID‐19 patients (45.82 pg/ml), but the differences were not statistically significant compared with the control group (36.32 pg/ml, *p* > 0.05). However, we observed a significant upward trend for SC and IL‐6 levels from mild disease (3760 ng/ml and 35.23 pg/ml, respectively) to severe disease (5700 ng/ml and 53.12 pg/ml, respectively) (Figure 1A,B). SC and IL‐6 levels without 20 samples from patients that were late sampled (described in Method section) did not show a difference in the significance of the results mentioned above (Figure S1A,C).

Our results showed that disease exacerbation positively associated to the elevated SC (*r* = 0.766, *p* < 0.0001) and IL‐6 (*r* = 0.433, *p* < 0.001). But regarding the difference in time of symptoms onset, we doubted whether SC and IL‐6 levels were affected by time. The regression model revealed that sampling time (from symptoms onset) did not affect SC (R^2^ = 0.017, *p* = 0.278) and IL‐6 (R^2^ = 0.007, *p* = 0.50) levels. A two‐way ANOVA also confirmed that mild to severe subgroups had significantly different SC and IL‐6 levels, regardless of how many days had elapsed since the disease onset. This issue was shown by the nonsignificant results of the main and interaction effects of sampling time on both SC and IL‐6 (Figure 1C,D).

As explained in Methods section, all patients were sampled when their disease was still active. Therefore, the above results proposed the possibility that SC and IL‐6 levels would be abnormal and/or higher before patients recovered. We found that all 8 patients (1 mild, 3 moderate, and 4 severe) who underwent resampling, at an interval of 6 d, showed clinically meaningful improvement. Notably, their levels of both SC and IL‐6 trended downward (Figure 1E,F), which were comparable to control subjects (Figure S1C,D).

### 3.3 Laboratory parameters with correlation to serum calprotectin

In Table 1, the results of other biomarkers in COVID‐19 patients are summarized compared to the control group as well as the COVID‐19 subgroups comparison. The absolute number of neutrophils (NEU) in COVID‐19 was not significantly different from the control (*p* = 0.620); however, there was a noticeable increase in severe compared with mild forms (*p* = 0.018). The absolute lymphocytes count (LYM) showed a significant decrease in COVID‐19 (*p* < 0.0001) irrespective of disease severity. Additionally, COVID‐19 was significantly associated with higher NEU%, neutrophil‐to‐lymphocyte ratio (NLR), lower LYM%, and platelet (PLT). These changes were more observed in severe forms of the disease. The lactate dehydrogenase (LDH) levels had highly increase in COVID‐19 (*p* < 0.0001) related to disease exacerbation (*p* = 0.009). Acute phase reactants, ESR, and CRP were found also significantly higher in COVID‐19 (*p* < 0.0001 and *p* = 0.002, respectively) regardless of disease severity.

**FIGURE 2:**
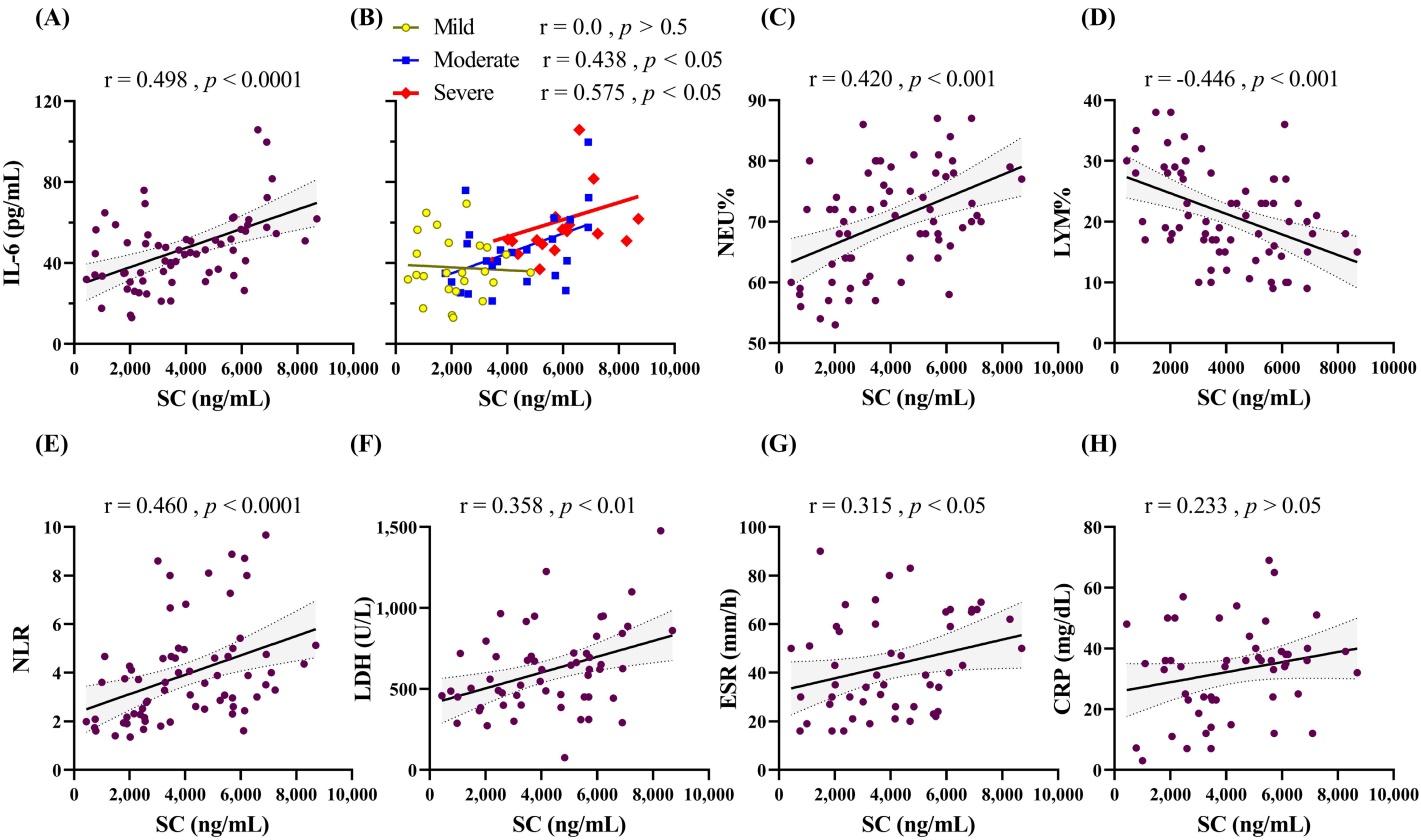
SC correlation with common biomarkers in COVID‐19. Spearman's correlation coefficients were calculated for (A) IL‐6, (B) IL‐6 concerning COVID‐19 severity, (C) NEU%, (D) LYM%, (E) NLR, (F) LDH, (G) ESR, and (H) CRP. The correlation coefficient (r) and *p*‐Values are shown above each plot.

SC demonstrated a positive correlation with IL‐6 (specifically with severe form), NEU%, NLR, LDH, ESR (Figure 2), and absolute NEU (*r* = 0.420, *p* < 0.0001). There was a negative correlation with LYM% (Figure 2D), and Hb (*r* = −0.277, *p* < 0.05). Even though the majority of them were significant, the strength of correlation was moderate (0.4 < r < 0.69) or weak (0.2 < r < 0.39). There was no significant correlation with CRP (Figure 2G), absolute LYM, and PLT.

### 3.4 Serum calprotectin rises as clinical status deterioration

**FIGURE 3:**
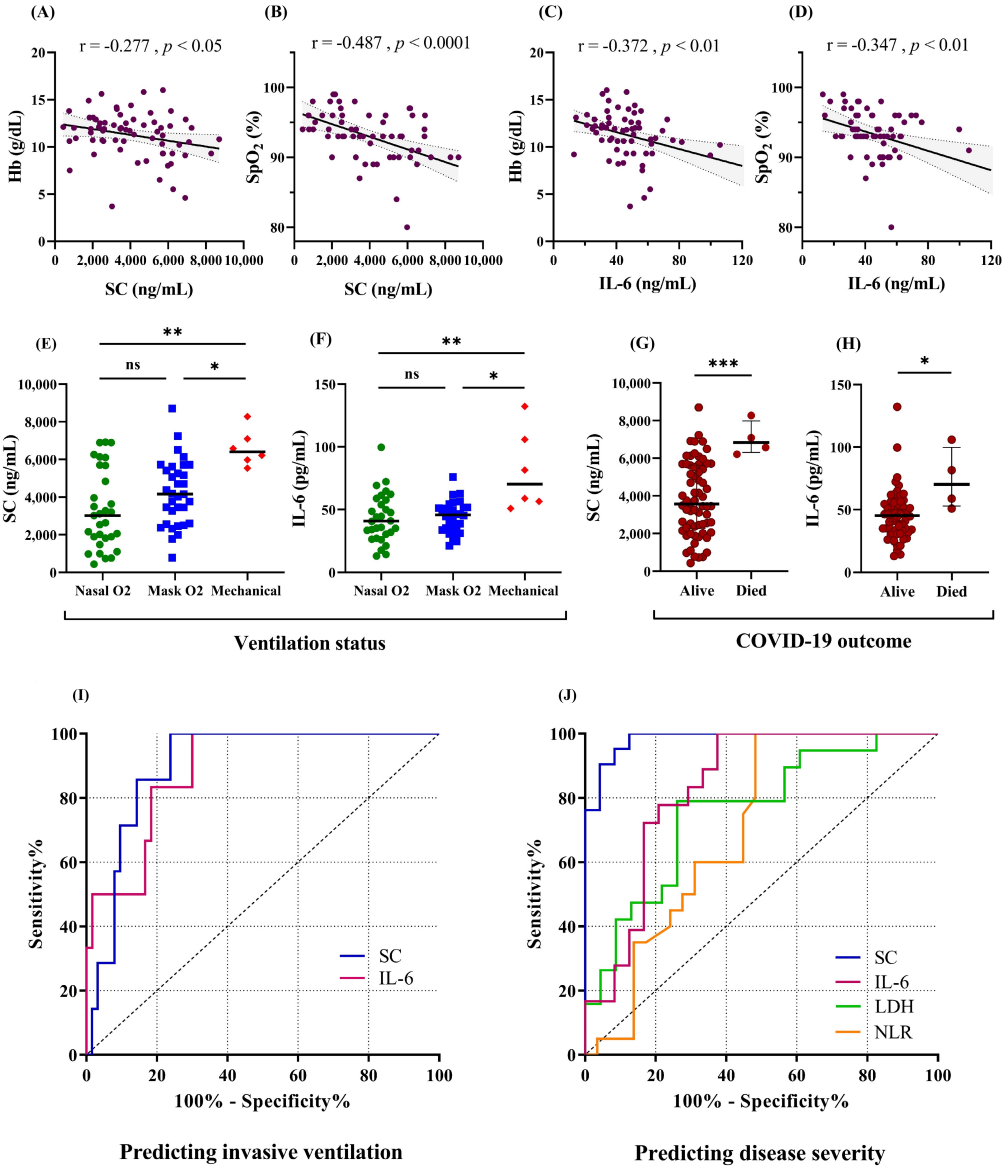
SC and IL‐6 relation to patients' clinical status. (A–D) The correlation of SC and IL‐6 with oxygenation status (Hb and SpO_2_). The correlation coefficient (r) and *p*‐Values are shown above the plots. SC and IL‐6 concentrations according to (E, F) the patients' ventilation status (nasal, mask, and mechanical) and (G, H) their survival status (alive or dead). Plots display all samples as dots, the median in the center line, and the *p*‐value of statuses pairwise comparison as *p < 0.05, **p < 0.01, ***p < 0.001, and ns (not significant) using Kruskal–Wallis test and Mann–Whitney test, respectively. (I, J) Receiver operating characteristic curves (ROC) identified the utility of inflammatory parameters to predict the need for invasive ventilation and severe illness of COVID‐19, respectively. Areas under the curve (AUCs) and *p*‐Values for invasive mechanical ventilation: SC = 0.903, *p* = 0.0005; IL‐6: 0.889, *p* = 0.002. AUCs and *p*‐values for COVID‐19 severity: SC = 0.984, *p* < 0.0001; IL‐6: 0.833, *p* = 0.001; HDL = 0.760, *p* = 0.004; NLR = 0.703, *p* = 0.017.

We found a significant negative correlation between SC and/or IL‐6 with oxygenation status (Hb and SpO2) (Figure 3A–D). Furthermore, patients requiring mechanical ventilation (6/76) had significantly higher levels of SC and IL‐6 compared with patients breathing with both conditions of noninvasive oxygen support (Figure 3E,F). In contrast, ESR, CRP, and LDH had no association with oxygenation status, as well as could not discriminate between invasive and noninvasive oxygen status (data not shown).

We also found a positive correlation between length of hospitalization (or treatment) with disease severity (*r* = 0.354, *p* < 0.01) as well as with SC (*r* = 0.476, *p* < 0.0001), but not with IL‐6 (*r* = 0.237, *p* = 0.06). Patients who passed away (4 of whom required mechanical ventilation) had far elevated levels of SC and IL‐6 than those who survived (Figures 3G,H).

Univariate analysis in the logistic model indicated no predictive value for demographic variables or comorbidities for mechanical ventilation and only age could predict the disease severity (Table 2). Only SC, IL‐6, and LDH were predictive of need for mechanical ventilation; however, LDH could not predict the need in multivariate analysis. The same analyses were performed to predict the severity progression. Both models showed similar patterns of significance for the three variables SC, IL‐6, and LDH. Regression coefficients of determination indicated that the logistic models were good fit and included parameters could explain 75% – 100% the probability of severe form of the disease (*p* < 0.0001), and 32%–64% probability of invasive ventilation requiring (*p* < 0.0001).

**TABLE 2:** Logistic regression for prediction of invasive ventilation and disease severity.

Variables	Invasive ventilation				Disease severity			
	Univariate analysis		Multivariate analysis		Univariate analysis		Multivariate analysis	
	OR (95%CI)	Sig	OR (95%CI)	Sig	OR (95%CI)	Sig	OR (95%CI)	Sig
**Age**	1.048 (0.990–1.110)	0.107	1.062 (0.989–1.140)	0.099	1.051 (1.009–1.094)	**0.015**	1.055 (1.007–1.106)	**0.024**
**BMI**	1.135 (0.957–1.345)	0.145	1.219 (0.988–1.504)	0.065	1.024 (0.920–1.140)	0.660	‐	‐
**Comorbidities**	197,813,240 (0.00‐)	0.998	‐	‐	2.571 (0.603–10.976)	0.202	‐	‐
**SC**	1.001 (1.000–1.002)	**0.010**	1.001 (1.000–1.002)	**0.036**	1.003 (1.001–1.004)	**0.001**	1.003 (1.022–1.171)	**0.009**
**IL‐6**	1.075 (1.022–1.130)	**0.005**	1.061 (1.010–1.114)	**0.019**	1.097 (0.993–1.212)	**0.005**	1.094 (1.010–1.210)	**0.010**
**LDH**	1.003 (1.000–1.007)	**0.038**	1.003 (0.999–1.007)	0.096	1.003 (1.001–1.006)	**0.019**	1.006 (1.002–1.011)	**0.005**
**NLR**	1.206 (0.856–1.699)	0.285	‐	‐	1.252 (0.951–1.649)	0.109	1.173 (0.877–1.569)	0.282
**CRP**	1.024 (0.969–1.081)	0.405	‐	‐	1.037 (0.995–1.081)	0.084	1.048 (0.999–1.100)	0.064
**ESR**	1.024 (0.978–1.071)	0.316	‐	‐	1.023 (0.988–1.060)	0.196	1.033 (0.993–1.074)	0.108

*Note*: Significant values (< 0.05) are bold. Multivariate analysis was not performed for the parameters whose univariate analysis significance was greater than 0.2. Thus, they were not included in the Table.

Abbreviations: CI, confidence interval; OR, Odd ratio.

Analysis of the ROC curves in Table 3 illustrated a high area under the curve (AUC) for SC and IL‐6 as predictors of requiring mechanical ventilation (Figure 3I). Furthermore, SC had a superior AUC for predicting the clinical severity (or deterioration of patients), with the highest sensitivity and specificity, compared with IL‐6, LDH, and NLR (Figure 3J). Other parameters did not show significant diagnostic values for respiratory status or clinical severity, so are not shown in the Table.

**TABLE 3:** Results of the ROC curve analysis.

Variables	Cut‐off	AUC (95% CI)	Sensitivity% (95%CI)	Specificity% (95%CI)	Sig	Youden's index
**Invasive ventilation**						
**SC**	> 5855 ng/ml	0.903 (0.825–0.980)	85.71 (42.13–99.64)	85.71 (74.61–93.25)	**0.0005**	71.42
**IL‐6**	> 56.54 pg/ml	0.889 (0.783–0.995)	83.33 (35.88–99.58)	81.67 (69.56–90.48)	**0.002**	65
**Disease severity**						
**SC**	> 3990 ng/ml	0.984 (0.958–1.000)	90.48 (69.62–98.83)	95.83 (78.88–99.89)	**<0.0001**	86.31
**IL‐6**	> 49.00 pg/ml	0.833 (0.582–0.845)	77.78 (52.36–93.59)	79.17 (57.85–92.87)	**0.001**	56.95
**HDL**	> 636.00 U/L	0.760 (0.613–0.906)	73.68 (51.21–88.19)	73.17 (58.07–84.31)	**0.004**	46.85
**NLR**	> 3.040	0.703 (0.557–0.849)	75.00 (50.90–91.34)	55.17 (35.69–73.55)	**0.017**	30.17

*Note*: Statistically significant (*p*‐Value <0.05) indicates by bold text. Any parameters with a non‐significant AUC were not included.

Abbreviations: AUC, Area under the ROC curve; CI, confidence interval; ROC, Receiver operating characteristic curves.

## 4 DISCUSSION

COVID‐19 has posed serious pressure on the health status and life of people worldwide.^22^ This pandemic respiratory viral disease has infected millions of cases and caused death in a wide range of targeted ages across the globe.^23^, ^24^ The highly rapid contagiousness of COVID‐19 and mortality due to its severity are the most worrying aspects of this pandemic. Therefore, early identification of patients who may evolve to severe or critical disease is a priority in dealing with COVID‐19 to improve the recovery rate and reduce mortality. Many studies have been conducted to distinguish the severity of COVID‐19 using various biological markers. Indeed, checking the calprotectin also has become valuable for prognosticating patients' status.^12^, ^25^ Moreover, considering the problematic nature of COVID‐19, patients need to be followed up through treatments.^26^ Hence, information on monitoring calprotectin levels over the disease course can provide us with deep insight into the prognosis of COVID‐19 severity or patients' outcomes.

Our results revealed that SC had a disease severity‐dependent response for COVID‐19 patients. SC content exhibited a well‐differentiated pattern in which it increased significantly with the exacerbation of disease clinical symptoms. This differentiation is crucial in patients' monitoring/safe management, taking important clinical decisions, and timely action.^27^ Inconsistent with us, a recent study carried out in Pune, India reported that calprotectin levels were not related to disease severity, rather its elevation was merely a function of SARS‐CoV‐2 infection.^28^ However, our results were following previous clinical studies on elevated calprotectin in severe SARS‐CoV‐2 infection, as well as the ability of calprotectin to distinguish between mild and severe forms of the disease.^11^, ^12^, ^13^, ^25^, ^29^, ^30^ Therefore, it can be argued that the SC increase likely indicated the severity of inflammation in COVID‐19.

Multiple markers were investigated in SARS‐CoV‐2 infection and disease severity. Our finding of increased NEU count in severe compared with mild patients was in line with earlier observations.^31^, ^32^, ^33^ Neutrophils are implicated as active perpetrators of inflammation and respiratory compromise in COVID‐19.^11^, ^13^ As regards the main content of neutrophils and monocytes is calprotectin, our results seem to corroborate the role of these cells in COVID‐19 severity. Interestingly, neutrophil infiltration from respiratory damaged cells induced by SARS‐CoV‐2 into the circulation of severe COVID‐19 cases has been discovered.^11^ Thus, SC increase could support NEUs activation related to COVID‐19 severe complications. Moreover, our data of marked LYM decrease and NLR increase in severe patients could confirm multiple reports of their predictive value for disease severity.^34^, ^35^ Other inflammation‐related parameters including IL‐6, LDH, ESR, and CRP revealed a significant increase in COVID‐19 patients compared with control cases. Howsoever, it is pertinent to note that none of the measured markers were found as proper as SC in differentiating disease severity, although there was a positive correlation between SC and intended markers.

SC appears to be a potential predictive biomarker. We observed that SC levels remained high in severe and moderate patients during hospitalization, despite the different days of disease onset and taking treatment. This observation may indicate a contiguous inflammatory status. On the other hand, decreasing the levels of inflammatory markers reflects attenuating inflammation and consequently an improvement in disease severity.^36^ Our further investigation showed that SC levels significantly reduced to normal in recovered patients and were comparable to control cases. A supportive study, conducted by Shrivastava et al. compared calprotectin after the onset of disease in a weekly manner. Their observations of increased calprotectin during the two‐first weeks and its significant decline in recovered patients are in‐line with our study. It was suggested that neutrophils remain activated up to 14 days after the onset of inflammation and return to resting state by 15–30 days post‐infection,^28^ which could support our observations. Of note, raised SC levels in our patients, whether severe or mild, were significantly decreased at follow‐up, while they recorded calprotectin decrease irrespective of disease severity.^28^ Though our results were significant, a larger population may be suggested to provide stronger evidence of SC follow‐up due to the relatively small numbers.

Close follow‐up of lung function along with calprotectin was recommended to reduce cytokine imbalance in those who may be potential candidates for early interventions.^25^ In this regard, Hb and SpO_2_ were outlined as important pulmonary‐related biomarkers in COVID‐19 patients, indicating lung damage as well as disease severity.^37^ Our results revealed that negative significant correlations were obtained between SC and Hb/SpO_2_. Additionally, there was greater content of SC in COVID‐19 patients who required invasive oxygen support as well as dead patients. In line with our findings, Shi et al. reported that calprotectin was significantly higher in severe cases requiring mechanical ventilation than in those who remained free of intubation.^13^ The need for invasive ventilation as well as the fatal outcome was associated with a further rise in calprotectin levels in patients with severe disease.^29^, ^38^ Although there are conflicting results of not significantly higher calprotectin in deceased as well as discharged patients compared to hospitalized patients when measured in serum.^39^ Monitoring SC levels may point to the disease duration of COVID‐19 cases, as patients who had severe disease with higher SC concentrations had to be treated for a longer period of time. Also, SC had a significant positive correlation with the length of hospitalization obtained here. Therefore, SC levels could predict the length of treatment.

We compared the features of SC with IL‐6 because controversial results were available. Since the beginning of the COVID‐19 pandemic, many studies have explored the predictive value of IL‐6.^27^, ^40^, ^41^, ^42^ In addition to confirming previous studies, this study found that SC has more prognostic value and clinical significance than IL‐6. Serum IL‐6 has been introduced as the best available biomarker for the severity of COVID‐19^43^; however, our findings showed a stronger positive correlation of increased SC with disease severity than IL‐6. Ducastel et al. reported that IL‐6 and calprotectin effectively could predict mortality but did not have the best prognostic performances to predict the deterioration of patients' conditions.^44^ Howsoever, in the present study, ROC curve analysis emphasized the superior potential of SC to discriminate severe cases from nonsevere reliably and decide for using mechanical ventilation. On the other hand, Silvin et al. uncovered that in severe COVID‐19 patients, calprotectin was the most elevated biomarker, while IL‐6 was relatively at a lesser extent among the other 23 tested inflammatory markers. Moreover, the reported concentrations of both markers^11^ were close to our results.

To our knowledge, this was the first result of IL‐6 evaluation that trended downward in recovered patients, like SC. As reported in the results, SC concentration increased notably by COVID‐19, but since IL‐6 did not significantly increase in COVID‐19 patients, its follow‐up may not be as an effective marker as SC for assessing disease recovery. This finding might be explained by the fact that calprotectin is upstream of the synthesis of inflammatory mediators, such as IL‐6, IL‐8, and tumor necrosis factor‐alpha (TNF‐α). Calprotectin binding to TLR4 and RAGE triggers the inflammatory cascade via nuclear factor‐kappa B (NF‐κB) translocation into the nucleus, itself overexpression induction, proinflammatory cytokines production, reactive oxygen species (ROS) generation as crucial inflammatory activators of granulocytes, and adhesion molecules synthesis contributing to inflammatory response amplification. In fact, calprotectin elevation is one of the first responses to an inflammatory condition.^44^, ^45^, ^46^ Therefore, we concluded that calprotectin likely accounts for the cytokine release syndrome triggering that determines COVID‐19 severity as SC clearly indicated the highest discrimination ability over other biomarkers for predicting poor outcomes in COVID‐19 patients with regard to our results.

While concluding that we included modest patients compared to the published clinical series because this study has conducted early in the course of the COVID‐19 pandemic peak. A larger series of patients may be needed to further accurately clarify the significance of biomarkers differences and their prognostic value between COVID‐19 and controls. Howsoever, the conclusion that elevated SC is an early marker of severe COVID‐19, as well as a risk indicator for disease progression, still appears to be strongly warranted. Next, we were able to correlate SC levels with neutrophils activation as our study contained complete hematological data.

## 5 CONCLUSIONS

The study data suggest that the activation of neutrophils assessed by the increase in SC and IL‐6 levels varied with disease severity. Our main finding was the more significant prognostic efficiency of SC in association with clinical status compared with IL‐6 and other biomarkers. SC appeared as an effective marker to predict the future status of COVID‐19 patients' follow‐up. Moreover, the strong association of SC with poor clinical outcomes further highlighted the potential role of this marker in disease progression. Our findings reinforce the idea that modulating the immune system can help to control the development of the disease. Therefore, urgent immunomodulatory therapy can require to be undertaken on priority.

## AUTHOR CONTRIBUTIONS

Hajar Shokri‐Afra: Designed the project, provided the grant, carried out experiments, drafted the manuscript, and critically revised the manuscript. Mona Moradi: Included patients in the work and contributed to experimental assays. Hadis Musavi: Contributed to draft the manuscript. Bahman Moradipoodeh: Substantial contributed to the conception or design of the work and critically revised the manuscript. Hemen Moradi‐Sardareh: Analysis or interpretation of data for the work and critically revised the manuscript. Arash Kazemi Veisari: Substantial contributed to the conception or design of the work. Ziaeddin Oladi: Included patients in the work and accountable for all aspects of the work. Mahboobe Ebrahimi: Substantial contributed to the conception or design of the work and critically revised the manuscript. All authors contributed in final approving the final manuscript to be published.

## CONFLICT OF INTEREST

All authors have disclosed that they do not have any potential conflicts of interest.

## Supporting information


Figure S1.

## Data Availability

Data available on request from the authors; The data that support the findings of this study are available from the corresponding author upon reasonable request.
