# Supplementary figures and images for: Serum calprotectin can indicate current and future severity of COVID‐19
Source: J Clin Lab Anal. 2022 Dec 16;37(1):e24809. doi: 10.1002/jcla.24809 (PMC9833977; doi:10.1002/jcla.24809)

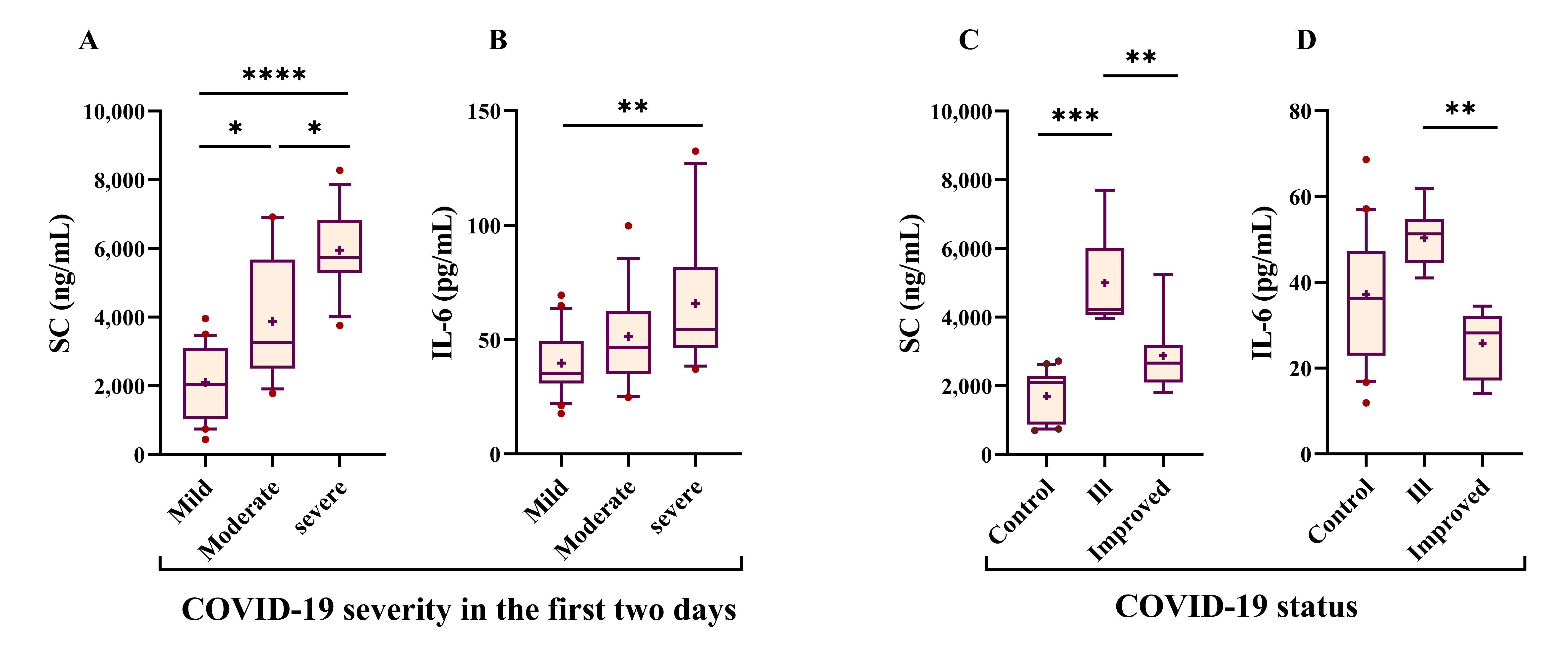

Supplement: Supplementary file 1 — Figure S1. [file JCLA-37-0-s001.tif]
